# Rubella and Its Relations to Scarlet Fever and Measles

**Published:** 1908-04-04

**Authors:** 


### Public Health and Hygiene.

### RUBELLA AND ITS RELATIONS TO SCARLET FEVER AND MEASLES.

Typical cases of scarlet fever and measles present little difficulty to the experienced eye; but the occurrence of atypical cases and of a minor disease of varying type, Rubella, which superficially, at least, simulates either scarlatina or morbilli, raises doubt, which frequently can only be allayed by the continued observation of the case.

On this ground, if upon no other, Rubella attains a degree of importance which is quite disproportionate to its position among acute diseases otherwise of serious character. At a recent meeting of the Epidemiological Section of the Royal Society of Medicine, Drs. E. W. Goodall and H. E. Corbin made two valuable communications on the nature and epidemiology of this disease. Rubella, German Measles, Rötheln Rubeola, Roseola, or Epidemic Rose Rash—to catalogue the various titles by which it has been known, must be regarded as definitely a specific infectious disease quite distinct from those which it resembles. The disease, according to Dr. Goodall, is feebly infectious, but there does not appear to be a consensus of opinion on this point. Dr. Corbin believes the period of infectiousness to be of short duration, reaching its maximum "during the short prodromal period of usually twenty-four hours, which occurs before the eruption is manifest, declining rapidly during the following twenty-four hours and disappearing entirely at the end of this period unless faucial catarrh persists."

It is possibly due to the brief duration of its infectiousness that the character of certain outbreaks has suggested feeble infectivity. Institutional outbreaks have frequently pointed to a very high degree of infectivity, and this may be accounted for by the fact that in such outbreaks there is a concentration of exposed persons during a brief period of exalted infectiousness. When, owing to removal of the patient, exposure of unprotected persons occurs only during the later eruptive stage, the affection would present the appearance of being feebly infectious. This view of the infectiousness of the disease possibly also accounts for the remarkable experience of Dr. Corbin at the London Fever Hospital, where, although cases both of Rubella and Measles are admitted into the same wards, "not one patient with either of these diseases has developed the other." The admission of cases to a hospital for purposes of isolation, and after the earlier, more highly infectious stage was past, would almost necessarily give different results to those which would be observed where an outbreak occurs among the inmates of an institution who are thus exposed in the earlier highly infectious stage of the disease.

Dr. Goodall gave a very concise account of the clinical features of the disease as he had observed them, and these are so important when the differential diagnosis of measles or scarlet fever arises that we offer no apology for quoting at length.

"*Prodromal Period.*—Usually none; if present, very short, seldom more than twenty-four hours. Of 85 cases which I saw last year, in 60 the rash was the first, or amongst the first symptoms. Other prodromes are sore throat, vomiting, enlargement of lymphatic glands, and moderate pyrexia. Less frequent are shivering, headache, giddiness, coryza, and pain in the back and limbs.

"*Rash.*—This usually commences on the face and scalp, as discrete pale pink spots, but not infrequently the spots come out on the face, trunk, and extremities simultaneously. When the face is first affected, the rash will disappear from it within twenty-four hours, and will then be seen on the trunk and upper extremities; lastly, the lower extremities are invaded. The rash involves the skin of the face right up to the lips. In most cases the rash becomes so confluent on the trunk and extremities the day after its appearance as to present a uniform pink or scarlet erythema, which is often punctate. As the rash has by this time disappeared from the face, the resemblance to mild scarlet fever is very striking. Sometimes the discrete spots fade and vanish without becoming confluent; less often they become confluent so as to form irregularly shaped macules, though the macules are not usually so large as those of measles. Still less frequently the rash takes the form of a scarlatiniform erythema on the trunk and limbs from the very commencement, avoiding the face. The duration of the rash is rarely longer than three days; often it is shorter.

"*Glands.*—The superficial lympathic glands are often moderately enlarged and tender. Those most commonly affected are the mastoid and posterior cervical, so that some stiffness of the neck results; but all may be implicated. They do not become matted together, and suppuration is extremely rare; I have never seen it myself. Of 67 cases last year in which a note was made as to the state of the glands, enlargement was observed in 52. In 18 cases several sets of glands were involved.

"*Pyrexia.*—In 41 cases observed all through the attack, the temperature rose above 99° F. in 15. In 44 cases admitted after the rash had come out, the temperature was above 99° F. in 20. The highest temperature recorded in these 85 cases was 102.8° F. Seldom is the temperature raised for longer than twenty-four hours.

"The *conjunctivæ* are often, but by no means always, infected. In only 29 of the 85 cases is a note made on this point—in 19 there was infection, in 10 there was not. The conjunctival affection causes itching and smarting of the eyes with lachrymation, and occasionally photophobia. Sometimes there is a slight watery discharge from the nose, with itching and sneezing. *Desquamation* may follow; usually it is slight and branny, but it may be profuse; rarely is it 'pinhole.'"

It was pointed out during the discussion that the clean mouth, the absence of Koplik's spots, and the invasion by the eruption of the circumoral region—rarely, if ever, noticed in scarlet fever—were features of the disease of great value in the differential diagnosis.

